# Proceedings of the American Dental Association

**Published:** 1872-10

**Authors:** W. H. Eames


### SELECTED ARTICLES.

### ARTICLE V.

### *Proceedings of the American Dental Association.*

Reported for *Missouri Dental Journal* by W. H. Eames, D.D S.

The American Dental Association met at Niagara Falls, August 6, 1872; President Cushing, of Chicago, in the chair. After the preliminary exercises, the report of the Committee on Physiology was called for, and the chairman not being ready to report, it was passed for the present, and a report on Pathology was read by Dr. Judd, of St. Louis. The report considered principally the etiological branch of the subject, and gave a resumé of the doctrines relating to the causation of disease, which are generally accepted by the profession at the present day. Beale's doctrines relating to the causation of contagious fevers were shown to harmonize with the doctrines of the most eminent German pathologists, though they were generally supposed to be radically different from the latter.

After discussion by Drs. Atkinson and Judd, the Association Adjourned until 8 o'clock in the evening.

EVENING SESSION—TUESDAY.

Minutes read and approved.

Dr. Judd's paper on Dental Pathology was called up.

Dr. Atkinson caused a blackboard to be placed in front of the President, for the purpose of illustrating his Report on Pathology. Not having the right kind of brains to understand the contents of the paper, the writer can give no synopsis of it, and will only say that it was the extreme of Atkinsonism.

MORNING SESSION—WEDNESDAY.

Dr. Smith, of ——, asked, through the Executive Committee, to show and explain his method of producing local anæsthesia.

Dr. B—— wanted to know something of its composition, and whether it was a patented article or not.

Moved that it be referred to a committee to examine its merits.

The Association laid the motion on the table.

Dr. Jack spoke in an earnest manner against listening, for a moment, to a proposition to exhibit any secret therapeutic article which is used by the profession. He was astonished that a body of this character should listen to it at all. He considered it a disgrace to do so.

The Committees on Microscopy and Histology were granted further time to report.

Dr. Dickerman read a report on Dental Chemistry. He considered all decay of the teeth chemical, and that dental chemistry was a sealed book to two-thirds of the profession. He urged its importance to dentists, and ranges it with surgery, physiology, &c., and thought that a better knowledge of chemistry would do away with many operations. There are more ignorant of science than of operations. Chemistry is needed, if we expect any length of time to be successful dentists. Soda should always be used after eating lemons. Chemical changes are always going on in the mouth, and we ought to know what they are. Children even should be taught them—in the office, by papers and books. He advocated the introduction of dental chemistry in school books.

Dr. Buckingham was called upon to speak on the subject, and said chemistry formerly was applied to dead matter alone. Now it is being tried to apply it to living matter.

Prof. Summers of the Alabama University, was invited by the society to address the meeting on chemistry, and said, cold and heat disturbs the forces which maintain the integrity of the teeth; alkalies do not combine, but seem to analyze the teeth; alkalies were more injurious than acids; alkalies were bad for digestion; acids are secreted in the mouth, and show that they are necessary for neutralizing the alkalies. Nonsense to try to educate the people. People will not have their stomachs educated; will eat what they please. Know of nothing in chemistry which would help to preserve the teeth. Glycercole of thymol is painful on the teeth; unites with albumen, and makes a nice covering, but the nerve cries out, and says it ought not to be there. Nitrous oxide is the coming anæsthetic; directs its influence on the cerebro spinal system of nerves—never on the sympathetic.

Dr. Atkinson asked if nitric acid could be made in the blood from nit. ox.

No one ever died from it. Does not give its oxygen to the blood. Does not operate on the *blood*—only on the nerves—not on the ganglionic system, except by *reflex* action. Chloroform acts on the nervous system, and decomposes in the blood also, and gives up its chlorine to the blood. Carbonic acid does not accumulate in the blood. The venous blood does not show more carbon in the blood. Perfect anæsthesia can be produced without any change in the blood; always pass nit. ox. through per sulph. iron and water many times. In making it, if white fumes are produced, it is not fit to be used.

A vote of thanks was passed to Prof. Summers.

Dr. McQuillen was opposed to this society being a mutual admiration society, but was in favor of this, as the Professor did not belong to the society.

Dr. Buckingham explained.

Dr. McQuillen quoted authorities, showing that anæsthetics produced its first action on the blood, and by diminishing oxygen, acted on the nerves. He experimented in this direction. Animal blood, or human, drawn under anæsthetics, was found that the blood corpuscles were not changed. Other authors had experimented with blood out of the body, and found different results. Approves of nit. ox.; is a good thing. Only by bad uses is it bad; kept a dog and a cat for thirty minutes, and a rabbit for over an hour, without death; thinks no case can be cited as a death from its use. Surgeons of this country and England are afraid of it. He is in favor of liquid nit. oxide. Thinks it will soon be universally used by the medical profession, as it is now by the dental.

Dr. Bogue took exceptions to Professor Summers in some ideas regarding thymol, acids, alkaline, and dental education. He *could* educate his patients, and had. There were no filthy mouths in his practice. Living body is the same as dead body in a chemical view—no vital force presiding over the operations of the body.

Dr. Atkinson. Glycerole produces pain, but not thymol. The glycerole is only a convenient mode of using it. Nit. ox., and all anæsthetics produced their effects by depriving the blood of oxygen; could not be shown a case different. Pain produced in applications of therapeutic agents to pathological conditions is right. Deprivation of oxygen is the only thing that produces anæsthesia; denies the assumption of the Professor, that no one could be injured by the use of nit. ox. Nit. ox. is not a cause; it is merely an occasion.

The Committee on Therapeutics was called upon to report.

Dr.—— offered a resolution that a paper be prepared upon the origin and condition of this Association, and given to the committee who have charge of the centennial anniversary of the Declaration of Independence.

Voted that the Executive Committee appoint the committee spoken of.

The Committee on Therapeutics reported:

Some improvement has been made since last session, mostly in topical remedies.

New remedy.—Oil of carraway; *carvatral*—U. S. Dispensary; colorless viscid oil; odor like that of creasote. It is carminative and stimulant in its action, and is best for sensitive dentine. Dry the dentine before application. Sensitive dentine is often caused by fluids of the mouth. By keeping the cavity shut up, sensation is reduced, especially by oxychloride of zinc, the latter is an escharotic, otherwise gutta percha would be as useful. Calcification is caused by stimulation by oxychloride.

Arsenous acid is discarded by the best in this Association. Tannic acid and chloroform, mixed in a paste, and kept in the cavity for a few days, is recommended. Sulphl. morphine has been recommended as an internal remedy.

Burring engines have been found to produce less pain in excavating dentine. Oxychloride zinc in exposed pulps has met with the greatest success in these cases, when protected by gold immediately, instead of postponing it; in young persons successful; in large pulp most successful; large proportion of healthy pulp to diseased; in exceptional cases, when there is not room for capping with gold, kill pulp at once. In pus secreting pulps too much risk; leaves it to others whose experience is more favorable in its use. Charity must be used by them towards those who know less.

C. W. Strong has invented a mode of bleaching by the use of some apparatus. It takes about fifteen minutes.

Dr. Atkinson used bottle and bent glass tube, black ox. manganese and hydrochloric acid.

Dr. Bogue's method of bleaching approved. Deprecates use of acids in removal of tartar.

Dr. Trueman approved of the paper, but dissented from some things. We find always that the teeth are decayed acid formations from foreign substance in the mouth. Fungi causes the acid condition of the mouth. Shut the cavity from the fluids of the mouth. Any kind of a filling will answer. Acid secretions are the cause of sensitive dentine. Has not had good success in capping with oxychloride of zinc, though not abandoned the practice. Uses the oxide of zinc, with creasote next to the pulp, and then oxycholoride. Bad constitution will always be against success.

Bleaching is successful almost universally: does not believe that a tooth can be bleached in fifteen minutes; must take three or four weeks; claims chloride of zinc and acetic acid as his own invention, seven or eight years ago; thinks the recommendation to use acids in cleaning teeth belongs to the dark ages of the world.

Dr. Butler opposes the use of chloride, or any acid fo bleaching teeth. Does not believe in bleaching teeth. Cuts out the discolored portion, fills the root, and leaves the cavity open, and it will bleach itself; or fill the crown with oxychloride of zinc, and cut out all but a thin layer of the oxychloride, and fill with gold.

Dr. Wetherbee. Sprits of camphor works very well, indeed; tubes permeated by the solution; can't say why that is better than alcohol alone. Uses oxychloride in capping pulps. Has good success; is surprised that Trueman has not had better success. The failure is the exception of rule in treatment of the pulp. Cannot save the pulps that have produced pus, or been disintergated. Don't believe it possible to help our patients in these bad cases. In cleaning teeth acids are entirely unnecessary; must be injurious to the surface of the teeth. Must be culpable if we use them. Has delicate and nice instruments to clean the teeth.

Dr. Crouse. Tried experiment; left teeth with bits of tartar on them in sulphuric acid, and the tartar was not removed. Used aromatic sulphuric acids. Use oxychloride for capping pulps, and succeeds in forty-nine out of fifty cases.

Dr. G. R. Thomas. I use oxychloride for saving pulps. I protect the pulps with thin paper, wet with carbolic acid. Glycerole of Thymol gives much pain in my practice. I have abandoned it.

EVENING SESSION—WEDNESDAY.

Dr. Carroll. I like Guillois' cement better than oxychloride of zinc for capping exposed pulps. Do not know what Guillois' cement is; may be oxychloride of zinc.

Dr. Shepard advocated the use of the mouth glass in examining and filling during the whole operation, and advocates sitting while operating.

Dr. Noble approved of the mouth mirror for constant work; while operating can work as well looking in the glass as into the cavity.

Dr. Abbot advocated amalgam in filling children's teeth; teaches children to take care of their teeth. Uses creasote on pulp for two or three days. Uses gutta-percha and wax in equal quantities, melted in to the cavity; never uses wax or sandarach varnish. Failures in treatment of living pulps is one of the seventeen; had found ossification of pulp or new dentine; prefers Smith's oxychloride; wipes out the cavity with creasote. Always fills front teeth from the front side; does not object to the showing of the gold; does not use rubber until ready to fill.

Dr. Crouse fills front teeth from the back side; always uses rubber dam in preparing teeth; never fills children's teeth with amalgam. No one does who is not lazy.

Dr. Abbot only uses amalgam when it seems impossible to use anything else. Thinks with Dr. Crouse, that tin is as good, if not better, than gold. Has seen fillings of tin in good condition for thirty years.

Dr. Wetherby, of Boston, in front teeth uses rubber dam, then wedge, and then thin file; then file from under side. No person with a level head would file from the front side.

Dr. Butler indorsed tin for children's teeth; can fill with tin better than gold; takes less time; does not fill as much as formerly; cuts away more. Uses amalgam sometimes in children's teeth.

Dr. Moore showed a central incisor which had been forced from the mouth by inflamation, caused by a rubber band in the process of regulating.

THIRD DAY—EVENING SESSION.

After the transaction of other business, on motion, a ballot was taken to determine the next place of meeting, and "Put-in-Bay," on Lake Erie, was finally selected.

The election of officers then took place, when Dr G. P. C. Hunt, of Indianapolis, was elected President; L. D. Shepard, of Boston, First Vice President: M. S. Dean, of Chicago, Recording Secretary, and as usual, Goddard, of Louisville, Ky., Treasurer.

FOURTH DAY—MORNING SESSION.

Reports were read by Dr. Thomas, of Detroit, on Etiology, and by L. D. Shepard, of Boston, on Dental Education.

The latter report favored endowed schools of dentistry connected with medical colleges, but with a distinct organization, &c.

The subject of dental education elicited considerable discussion.

Dr. C. E. Francis read a report on Operative Dentistry, which was discussed by various members, and after some other reports and the transaction of the necessary business, the Association adjourned to meet at Put-in-Bay, Ohio, on the 1st Tuesday in August, 1873.

---

